# An Unusual Cause of Foot Drop: Peroneal Extraneural Ganglion Cyst

**DOI:** 10.12669/pjms.324.9998

**Published:** 2016

**Authors:** Murat Zumrut, Mehmet Demirayak, Ahmet Kucukapan

**Affiliations:** 1 Murat Zumrut, MD. Assistant Professor, Department of Orthopaedic Surgery, Medical Faculty, Mevlana University, Konya, Turkey; 2 Mehmet Demirayak, MD. Assistant Professor, Department of Orthopaedic Surgery, Medical Faculty, Mevlana University, Konya, Turkey; 3 Ahmet Kucukapan, MD Assistant Professor, Department of Radiodiagnosis, Medical Faculty, Mevlana University, Konya, Turkey

**Keywords:** Ganglion cyst, Peroneal nerve palsy, Foot drop

## Abstract

Peripheral neuropathies caused by ganglion cysts are quite rare, especially in the lower extremities. The case of a 64-year-old male with a 2-day history of foot drop and tenderness in the region of the left fibular neck is presented. Physical examination and electromyogram findings verified peroneal nerve palsy. Ultrasonography showed cystic mass localized proximal of the peroneal muscle structures. Magnetic resonance imaging revealed a cystic-appearing mass around the fibular neck that compressed the common peroneal nerve. Surgical excision and ligation of the cyst pedicle were performed. The pathology reports confirmed the diagnosis of a ganglion cyst. The patient regained full function within two months of the surgery. Early sensory symptoms before foot drop should be considered as an indication of surgical excision to prevent delayed damage. Ligation or electrocoagulation of the cyst pedicle should be a part of surgical procedure to avoid recurrences.

## INTRODUCTION

A ganglion cyst is a benign mass with a collagenous fibrous capsule surrounding high-viscosity mucinous fluid. Ganglions may arise from the joint capsule or sheath of a tendon and peripheral nerves. Although ganglionic cysts are very common lesions, they may rarely cause compression neuropathy. In the upper extremities, ganglia have been described to cause compression of the ulnar nerve in Guyon’s canal, in the cubital tunnel or of the median nerve at the carpal tunnel. Involvement of the lower extremities is much less common,^1^ and the compression of the peroneal nerve at the level of the knee and proximal tibiofibular joint has been described scarcely.^1^-^4^ In this report, a patient with common peroneal nerve palsy due to a ganglion cyst is presented as a rare case in the light of literature.

## CASE PRESENTATION

A 64-year-old male farmer presented to our outpatient clinic with a 2-day history of foot drop and tenderness in the region of the left fibular head. He complained of mild pain radiating from the knee to the ankle and numbness affecting the dorsal aspect of his left foot. The patient reported no history of trauma. In his examination, there was no evidence of degenerative disc disease of the lumbar spine. Careful physical examination revealed nebulous soft swelling below the fibular head. There were no signs of tibialis anterior or peroneal muscle atrophy. However, there was weakness in foot eversion, ankle dorsiflexion, and great toe extension. Plantar flexion and foot inversion were normal. We also found diminished sensation in the first web space of the left foot and positive Tinel’s sign near the fibular head.

**Fig.1:**
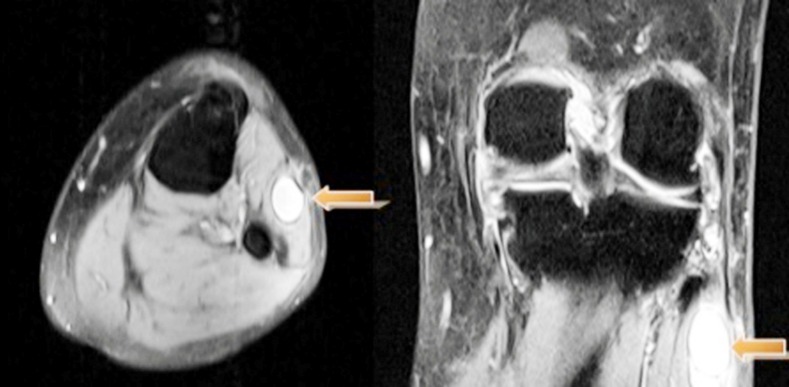
Proton density-weighted fat-saturated axial and coronal magnetic resonance images show close relationship of the cystic lesion with the peroneal nerve.

Electromyography (EMG) findings demonstrated denervation of the anterior tibial and peroneus muscles. No abnormality was found in the conduction velocity of the tibial and sural nerves. Radiographs of the knee showed normal findings. Ultrasonography (USG) revealed the 38 × 21 × 16 mm cystic mass localized between the muscle structures. Magnetic resonance imaging (MRI) revealed the presence of a fluid-filled mass, likely presenting a ganglion cyst, around the fibular neck, which compressed the common peroneal nerve (Fig.1).

Surgery was performed with a lateral approach under a pneumatic tourniquet. The cyst was seen to arise from the anterior aspect of the proximal tibiofibular joint with a distinct pedicle. It was compressing the peroneal nerve at the level of the fibular neck (Fig.2A). Careful dissection was carried out under loupe magnification to preserve all nerve branches, and the cyst was completely removed after its pedicle was ligated (Fig.2B). The histopathologic evaluation confirmed the diagnosis of a ganglion cyst.

**Fig.2A:**
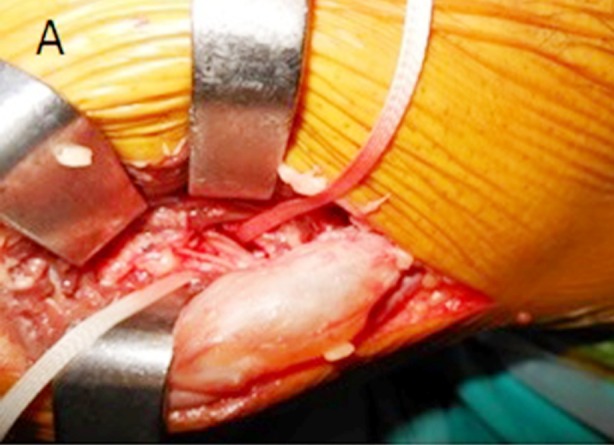
Intraoperative photograph showing relative positions of the peroneal nerve and ganglion.

**Fig.2B:**
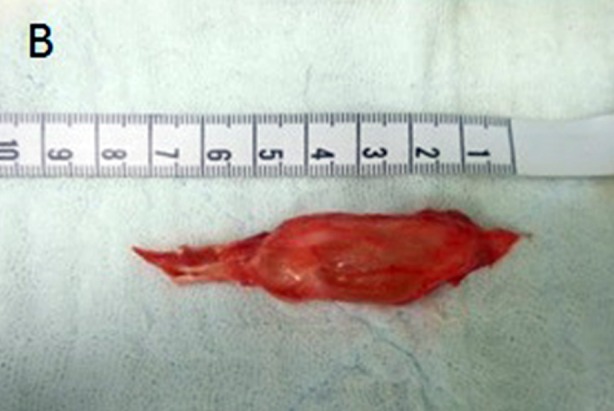
Ganglion with its pedicle completely excised.

Postoperatively, the patient was mobilized with an antifoot-drop peroneus splint and treated with intensive physiotherapy. In the early days after surgery, he had no complaints of pain. Sensory loss and motor function were almost fully recovered 2 months after operation. Two years after the operation, there was no recurrence.

## DISCUSSION

Although ganglion cyst is the most common cause of non-traumatic peroneal nerve palsy, peroneal nerve palsy caused by a ganglion is a rare entity. Historically, Brooks described three cases of peroneal nerve compression due to ganglia in 1952.^5^ In 1965, Stack reported 9 cases of compression of the peroneal nerve.^6^

Ganglion cysts compressing the peroneal nerve may be extraneural or intraneural. Most of the ganglionic cysts described in the literature causing peroneal nerve compression were of an intraneural type.^2^ In the presented case in this report, compression of the peroneal nerve was due to a rarely encountered extraneural cyst.

The pathogenesis of these cysts has been the subject of controversy, but the hypothesis of articular (synovial) origin from the proximal tibiofibular joint seems to be most likely.^2^ In the formation of intraneural cysts, degenerative synovial joint fluid passes through a capsular defect into an articular branch of the peroneal nerve and dissects into the parent nerve. However, extraneural cysts are connected to joints through capsular tears, which are presumably located separate from an articular branch. Their connection is therefore via a non-neural pedicle.^7^

Electrodiagnostic studies can help localize the lesion and determine the severity of the neuropathy. Either USG or MRI can be used as a diagnostic method. USG is an effective, cheap, and non-invasive technique to identify the cyst. USG may also be useful in distinguishing cysts from solid tumors, but it provides limited knowledge about the surrounding tissues. MRI may be used to identify the anatomical relation of the cysts to the joint and surrounding structures, but it is more expensive than USG. A combination of USG and MRI may be helpful in ensuring accurate diagnosis.^2^ EMG, USG, and MRI were performed in the present case.

L4/L5 root pathologies should be considered in the differential diagnosis. If the ganglion cyst is concealed by swelling, diagnosis may be difficult. However, positive Tinel’s sign and tenderness around the fibular neck can be keys to differentiate the root pathologies. Meticulous neurological and neurophysiological examination must be done to avoid a misdiagnosis.^8^

Various treatment regimens have been described over the years. Some selected cases were reported as being treated successfully with either simple cyst aspiration or cyst aspiration with corticosteroid injection.^9^,^10^ Because of the extensive branching pattern of the peroneal nerve in the area of the fibular head, needle aspiration carries a substantial risk for nerve damage.^1^ In addition, high recurrence rate and low patient satisfaction make this method less favorable. In our opinion, it should be used for the treatment of patients who refuse surgery.

Currently, the accepted treatment method of the ganglion cyst is marginal excision.^1^-^3^ In addition to marginal excision, ligation or electrocoagulation of the cyst pedicle should be a part of the surgical procedure to avoid recurrences. Some authors stress the importance of ligation of the articular branch of peroneal nerve.^11^,^12^ However, we think that excision with ligation of the cyst pedicle is sufficient in the treatment of extraneural cysts. Because the connection with the joint is via a non-neural pedicle in extraneural cysts, ligation of the articular branch is unnecessary. It seems necessary only in the treatment of intraneural cysts to avoid recurrence.

Furthermore, there is no consensus about the timing of surgery. Greer-Bayramoglu et al. recommended open decompression of the peroneal nerve between the third and fourth months if symptoms persist or recovery is incomplete.^1^ However, Fansa et al. recommended excision even if there are no neurological symptoms.^10^ In our opinion, early sensory symptoms should be considered as an indication of surgical excision to prevent delayed damage.

In conclusion, peroneal nerve palsy caused by an extraneural ganglion is a rare condition. A combination of EMG, USG, and MRI is useful for accurate diagnosis. L4/L5 root pathologies should be considered in the differential diagnosis. Early marginal excision of the cyst with ligation or electrocoagulation of its pedicle is sufficient for satisfactory outcomes.
